# The journey of patients with musculoskeletal complaints in Europe: a cross-sectional European survey

**DOI:** 10.1007/s00296-025-05863-x

**Published:** 2025-04-18

**Authors:** Magali Wagner, Teresa Otón, Felix Muehlensiepen, Karin Stratingh, Estíbaliz Loza, Rachel Knevel, Loreto Carmona

**Affiliations:** 1 Center for Health Services Research, Faculty of Health Sciences Brandenburg, Brandenburg Medical School Theodor Fontane, Rüdersdorf bei Berlin, Germany; 2 Instituto de Salud Musculoesquelética, Madrid, Spain; 3 Dutch Arthritis Society, Amsterdam, The Netherlands; 4 Department of Rheumatology, Leiden University Medical Center, Leiden, The Netherlands

**Keywords:** Healthcare access barriers, Patient journey, Patient experience, Rheumatic and musculoskeletal diseases, surveys and questionnaires, Surveys and questionnaires

## Abstract

Rheumatic and musculoskeletal diseases (RMDs) are highly prevalent and place a significant socioeconomic burden on healthcare systems. However, their diagnosis and management remain suboptimal. This study aimed to analyze healthcare-seeking behaviors, key touchpoints, access barriers, and diagnostic pathways for individuals experiencing initial or progressive symptoms of RMDs across European countries. Understanding these differences is crucial for improving early access to specialized care. A cross-sectional online survey was conducted with 141 participants from seven European countries, including 67 rheumatologists and 39 general practitioners (GPs). The survey assessed initial healthcare-seeking behaviors, delays in diagnosis, and perceived barriers to specialized rheumatology care. Descriptive and inferential statistical methods were used for data analysis. The survey indicated that individuals experiencing RMD symptoms primarily seek information through internet research and GP consultations. Despite their role as primary gatekeepers, GPs’ knowledge of RMDs was generally perceived as moderate to low. Significant disparities in access to rheumatological diagnostics, time to diagnosis, and treatment, coupled with organizational barriers between primary and specialist care, were reported across most countries. Spanish participants reported the longest diagnostic delays, while Swedish respondents experienced the shortest. Additionally, access to sacroiliac MRI was limited in Hungary and Spain, whereas glucocorticoids were widely available across all countries according to the participants. The study also revealed that early arthritis clinics were most accessible in the UK from the participants’ perspectives. Significant variations in healthcare access for patients with RMDs persist across Europe. Strategies to enhance early detection, including GP education and improved specialist accessibility, are essential to optimizing patient outcomes.

**Supplementary Information:**

The online version contains supplementary material available at 10.1007/s00296-025-05863-x.

## Introduction

Rheumatic and musculoskeletal diseases (RMDs) include over 200 conditions affecting joints, the skeleton, and connective tissues [1, 2]. Their causes include genetic predisposition, environmental, metabolic, and mechanical factors, and immune disorders [1, 2]. In Europe, over 120 million people suffer from RMDs, regardless of age or gender, and they have become a leading cause of disease burden [3]. According to the World Health Organization (WHO), as the global population ages, the prevalence of RMDs is expected to increase [4]. This is also due to higher obesity rates, inactivity, and poor dietary habits, which can harm the musculoskeletal system [3, 5].

Each RMD can manifest in symptoms, including musculoskeletal (MSK) pain, joint swelling or stiffness. A group of RMDs, collectively referred to as inflammatory rheumatic diseases (IRDs)—including rheumatoid arthritis (RA) and spondyloarthritis (SpA)—may have systemic involvement, affecting many organs and systems. Reduced mobility, increasing disability, fatigue, and psychological effects, such as depression, are intimately related to RMDs and result in impaired quality of life [2, 3, 6–8]. They also result in a significant number of doctor visits, prolonged periods of work disability, and even premature mortality [2, 3]. Consequently, IRDs incur substantial economic costs, including expenses associated with disease-specific treatments, reduced productivity or early retirement [3, 5, 8, 9].

The diagnosis of RMDs is often based on a combination of clinical examination, imaging and laboratory analyses [10]. However, not all forms of RMDs are diagnosed at early disease stages [2]. Concretely, outcomes in IRDs are markedly improved by reaching rheumatology within a window of opportunity [11]. Studies reveal a wide variation in time to diagnosis for RA, ranging from 1 month to 10 several years, with delays over 12 months associated with greater radiographic structural damage at follow-up in RA patients [12–16]. Gender, ethnicity, primary care physician’s knowledge, and diagnostic availability contribute to these delays [16–23]. Similarly, treating RMDs is complex. It usually requires a multidisciplinary approach, including drug therapy, physiotherapy and, sometimes, surgery [10].

Despite their high prevalence and impact, RMDs remain underdiagnosed and undertreated. Few professionals are adequately trained to assess, manage, and treat these conditions properly [24]. Some research and health service groups are developing solutions for this public health issue. One such group is the SPIDeRR (Stratification of Patients using Advanced Integrative Modelling of Data Routinely acquired for diagnosing Rheumatic complaints) consortium [25]. SPIDeRR aims to disentangle the complexities of early diagnosis of RMDs, discriminating as soon as possible the specific disease by considering the full spectrum of factors influencing patients’ symptoms. Besides, SPIDeRR aims to develop novel high-throughput methods to analyse multimodal clinical and biological data, generate supervised machine learning models for patient stratification, and integrate clinical and biological data to expedite diagnosis and personalised therapy choices.

To facilitate the future implementation of the SPIDeRR tools, a real-time, reliable European rheumatic patient journey map needed to be generated. For this purpose, several sub-studies were designed, including a European survey to identify the differences across countries on the health system characteristics and touchpoints once a person starts with MSK symptoms.

## Methods

We designed an online, cross-sectional survey targeting healthcare professionals and citizens with or without RMDs.

Brandenburg Medical School’s institutional review board approved the study protocol and materials (Date 13.02.2024, Reference: 167122023-BO-E).

This manuscript adheres to current recommendations on survey designing and reporting [26].

### Survey development and content

An international panel of experts, including experienced scientists, rheumatologists, a patient, and a medical student, developed the survey based on previous documentation on healthcare systems and a systematic review of patient journeys in RMDs [27]. The questionnaire design was informed by similar validated surveys assessing healthcare access and patient pathways in chronic disease management. The initial draft was revised three times following expert feedback to improve clarity, relevance, and comprehensiveness.

The survey consisted of three separate questionnaires. Two were tailored for healthcare professionals—general practitioners (GPs) and rheumatologists—and the third targeted citizens with and without diagnosed RMDs, whom we will call collectively ‘users’ for simplicity [28]. The forms included multiple-choice questions, matrix scales, drop-down menus, and comment boxes. They were designed to take less than 15 minutes to complete and were available in English.

All surveys were structured as follows: country of residence/practice, sociodemographic/practice questions (3–6 items), access to healthcare (4 items), care pathways (3–6 items), IT systems (2 items), and finally, a section for respondents’ perceptions on organisational aspects (7 items). The questionnaire for rheumatologists was the longest, with 23 items, and the one for citizens was the shortest, with 13 (All three surveys are available as supplementary material in Appendices 1, 2, and 3).

To ensure face validity and user-friendliness, the questionnaire underwent pretesting with a sample of 5 rheumatologists, 5 general practitioners, and 10 users, who provided feedback on clarity, coherence, and ease of completion. Adjustments were made to refine wording and improve response flow.

The survey was uploaded in a licensed SurveyMonkey account, a cloud-based platform and tool that allows users to create, distribute, and analyze surveys.

### Participants, recruitment, and sample size

The desired profile for participants was to be a rheumatologist, a GP, or a user of health services from the SPIDeRR countries (Germany, Greece, Hungary, the Netherlands, Spain, Sweden, and the United Kingdom) with or without RMDs. All should be adults and be able to understand English.

Recruitment was done through the SPIDeRR researchers’ networks using snowball sampling. We asked consortium members to propose at least one representative contact from the target groups in their country. The contacts were then informed about the objectives, content, and length of the survey and asked for their consent to participate before sending them the link. No incentives, such as money or subject hours, were provided.

The target sample size was 280 participants: 20 users (10 with and 10 without an RMD), 10 GPs, and 10 rheumatologists from each of the 7 countries. The sample per country was deemed sufficient as the questions were mainly about the objective characteristics of the health care systems, not opinions or personal data.

### Survey launch, data collection, and analysis

The three surveys were uploaded to the SurveyMonkey© platform. The authors tested and refined the survey to ensure user-friendliness. They also adverted that although the original language was English, internet browsers could automatically translate the survey into other languages. Links to the survey were sent to the preregistered participants; in countries where recruitment was not ideal, the links were sent to the researchers, together with information sheets for them to disseminate freely. The survey was active from April 20th to May 15th, 2024. Submission of the survey constitutes the participant’s consent to the survey.

Descriptive statistics and analysis of variance were used to analyse the distribution of responses and compare data across countries and groups. Missing data were not imputed.

Weighted scores were created to synthesise the responses to some questions with various response levels. A “Difficulty” score was calculated as a weighted sum of access scores, with “very difficult” responses multiplied by 5 and “very easy” by 1, with all other scores in between. Likewise, an “Experienced delay” score was calculated as a weighted sum of scores, with “More than 12 months” responses in the time to treatment multiplied by 5 and “Less than 1 month” by 1, with all other scores in between. Finally, an “organisational score” was calculated as a weighted sum of scores, with “very well [organised]” responses multiplied by 5 and “very poorly” by 1, and all other scores in between.

After many questions, open text fields allowed the respondents to clarify their responses. These are presented in the corresponding section of the results.

## Results

The survey sample comprised 141 users (142 responded, but 1 user from Finland was excluded due to not representing a target country), 67 rheumatologists, and 39 GPs. The sample was balanced across countries and participant groups (Table 1).

**Table 1:** Sample of surveys per country participant groups*

Country	Users (*n* = 141)	Rheumatologists (*n* = 67)	GPs (*n* = 39)	Total (%)
Germany	17 (12)	14 (21)	6 (15)	37 (26)
Greece	13 (9)	6 (9)	8 (21)	27 (19)
Hungary	23 (16)	8 (12)	2 (5)	33 (23)
Netherlands	13 (9)	5 (8)	8 (21)	26 (18)
Spain	20 (14)	21 (31)	8 (21)	49 (35)
Sweden	36 (26)	7 (11)	6 (15)	47 (33)
United Kingdom	19 (14)	6 (9)	1 (3)	26 (18)

*Cells represent the number of participants and percentage (%) per group (column)

Abbreviations: GPs, general practitioners

Among the respondent users, 64 (45%) had a confirmed diagnosis of an RMD (osteoarthritis [OA] *n* = 10, RA *n* = 9, SpA *n* = 5, psoriatic arthritis *n* = 5, and all others in frequency under 2). Of the 141 users, ninety-one users (64.5%) were women, and 121 (85%) were aged between 30 and 50 years. Most citizens considered themselves at a medium or high socioeconomic level (*n* = 128; 91%), whereas 4% were very high and 4% low (Supplementary File; Table S1).

Most rheumatologists worked in the public sector (*n* = 60; 89.6%) in a practice group or centre, sharing the pool of patients (*n* = 47; 70.2%). The gender and working experience were well balanced across the countries. The GPs were also mainly from the public sector (*n* = 36; 94.7%), from urban settings (*n* = 30; 79.0%), and practised primarily in a practice group or centre, not sharing the pool of patients (*n* = 24; 63.2%). Slightly more GPs were women (*n* = 26; 68.4%) and were balanced in terms of years of practising (Supplementary File; Table S1).

### Seeking help for an MSK problem

When participants were asked about how likely people with MSK complaints would be to use different pathways to seek help in their countries, the responses were similar across groups and countries, with searching the internet and going to the GP as the most likely touchpoints (Table 2).

**Table 2:** Perceived frequency (%) of people with MSK symptoms seeking help through different healthcare touchpoints from the perspective of users, gps and rheumatologists from 7 European countries, in order of frequency

Touchpoint or help-seeking behaviour	Users	GPs	Rheum
Go to GP	60	76	63
Search the internet directly (Google or a similar search engine)	53	59	47
Go directly or try to get an appointment with Rheumatology	33	42	67
Use a symptom checker**	26		
Go to Physiotherapy	25	24	6
Go directly or try an appointment with Orthopaedic Surgery/Traumatology	16	55	49
Go to Rehabilitation	13	13	11
Use AI, like ChatGPT**	6		
Go to a Sports Doctor	6	13	6
Go to an Occupational therapist	5	6	3
Go to a Pain unit	4	9	6
Check social media (TikTok, Facebook, Instagram, etc.)**	3		
Go to a Spine Clinic	3	9	13
Go to Balneotherapy	2	3	0
Visit a specialist nurse		3	2
Visits a psychologist (specialist in pain)		3	2

*Cells represent the proportion (%) of responses “Very likely”. Those left blank were not asked to the specific group

**These options were not offered to rheumatologists and GPs because we were more interested in the responses of nonmedical participants

Abbreviations: GPs, general practitioners; Rheum, rheumatologists; AI, Artificial Intelligence

### Primary care touchpoint

The survey investigated the role of the GP as a gatekeeper, the level of management of RMDs, and other aspects related to staying in primary care without a referral to specialised care.

#### GP’s gatekeeper role

Rheumatologists and GPs were asked how critical GPs are in referring patients to rheumatology (gatekeeping function). Table 3 shows the percentage of responses of rheumatologists and GPs by country to whether GPs mandatorily provide access to rheumatology. In Greece, GPs do not act as gatekeepers, which is the norm in the UK, Spain, and the Netherlands. In other countries, the responses of GPs contradict the rheumatologists’ (Hungary) or are variable. This latter may have to do with different pathways to rheumatology, like via emergency or other specialists or directly.

**Table 3:** Frequency (%) of gps functioning as gatekeepers in 7 European countries

	DE	HE	HU	NE	ES	SE	UK	Total
**% responses from rheumatologists**								
Yes	14	0	38	60	60	57	33	39
It depends	0	0	38	40	20	14	50	20
No	86	100	25	0	20	29	17	41
**% responses from GPs’**								
Yes	60	0	100	86	71	100	100	64
It depends	20	0	0	14	29	0	0	12
No	20	100	0	0	0	0	0	24

*Cells represent the percentage of answers per country

Abbreviations: DE, Germany; HE, Greece; HU, Hungary; NE, Netherlands; ES, Spain; SE, Sweden; UK, United Kingdom; GPs, general practitioners

#### GPs’ knowledge of RMDs

Rheumatologists and GPs were asked how knowledgeable they think GPs are about RMDs in their country (Supplementary File; Table S2). In general, knowledge of RMDs is perceived as moderate, being the largest in Sweden and the lowest in Hungary. The responses from rheumatologists and GPs are similar in each country.

#### GPs’ access to tests, medications and health care services related to RMDs

Rheumatologists and GPs were asked about access to laboratory and imaging tests in primary care. They were also asked whether GPs could prescribe medications commonly used in rheumatology, such as disease-modifying drugs (DMARDs) and glucocorticoids, to treat IRDs. Finally, rheumatologists and GPs were asked about access to other healthcare professionals with experience in RMDs or their complications directly from the primary care level (Table 4).

**Table 4:** Access at the primary care level to specific tests used in the differential diagnosis of RMDs, rheumatological treatments, and healthcare professionals in 7 European countries

	DE	HE	HU	NE	ES	SE	UK	Total
**Rheumatological tests**								
% responses from rheumatologists								
Rheumatoid factor	93	100	63	100	95	100	100	93
ACPA	93	100	0	80	52	100	83	93
HLA B27	93	100	0	80	57	100	67	93
Hands and feet X-rays	86	100	63	100	90	100	100	86
Sacroiliac MRI	79	100	13	60	29	100	67	79
% responses from GPs								
Rheumatoid factor	83	88	50	88	88	67	100	82
ACPA	50	75	0	88	13	67	100	56
HLA B27	33	63	0	75	25	50	100	49
ANA	33	88	0	88	88	67	100	72
Acute phase reactants (ESR, C-RP)	50	75	50	88	75	67	100	72
Hands and feet X-rays	50	88	100	88	88	67	100	79
Sacroiliac MRI	50	50	0	50	13	67	0	41
**DMARDs and glucocorticoids**								
% responses from rheumatologists								
Methotrexate	100	100	13	0	50	14	67	54
Sulfasalazine	100	100	38	20	50	14	67	58
Hydroxychloroquine	100	100	38	80	50	14	67	63
Glucocorticoids	100	100	88	100	100	100	100	98
% responses from GPs								
Methotrexate	67	63	0	13	38	17	0	36
Sulfasalazine	50	25	0	25	50	17	0	31
Hydroxychloroquine	50	63	0	13	38	0	0	31
Glucocorticoids	67	88	100	75	88	50	100	77
**Healthcare professionals**								Total
% responses from rheumatologists								
Physiotherapists	100	83	38	100	80	100	100	85
Psychologists	85	33	25	100	65	100	100	71
% responses from GPs								
Physiotherapists	83	25	0	88	88	67	100	67
Psychologists	67	25	0	75	38	67	100	51

Abbreviations: DE, Germany; HE, Greece; HU, Hungary; NE, Netherlands; ES, Spain; SE, Sweden; UK, United Kingdom; GPs, General Practitioners; ACPA, Anti-cyclic citrullinated peptide antibody; HLA B27, a specific gene variation for spondyloarthritis; ESR, erythrocyte sedimentation rate; C-RP, C-reactive protein; ANA, antinuclear antibodies; MRI, magnetic resonance imaging

The most accessible tests at the primary care level, from the responses of rheumatologists, are the rheumatoid factor, antibodies against the citrullinated peptides (ACPA), and HLA B27. Hungary and Spain had the lowest access to rheumatological tests in primary care. Sacroiliac MRI is the test with the lowest access in general.

Glucocorticoids are widely available across the target countries, while DMARDS are available in Germany and Greece but not so much elsewhere.

Access to physiotherapists in primary care was markedly low in Hungary compared to other countries. Access to psychologists was substantially lower in Hungary, Greece, and Spain than in other countries. The perceived access to other services varied mainly by service and less by the country or the stakeholder respondent (Supplementary File; Table S3). The professionals with the lowest perceived access were specialist nurses and occupational therapists in the public and private sectors, except in the UK, and balneotherapy in Hungary. There was some discordance between stakeholders regarding how large they perceived the access, but generally, it was very consistent, reflecting actual access or lack thereof. Most participants in all countries reported access to public and private primary care, orthopaedics and rheumatology. However, fewer participants reported access to private primary care in Hungary (44%, 50%, 0%), private orthopaedics in Sweden (30%) and the UK (56%), and private rheumatology in the Netherlands (22%, 50%, 60%) and Sweden (50%). Access to public physiotherapy is lower in Spain (33%) than private physiotherapy (93%); all other participants assume a high level of access in their countries. Occupational therapy is less accessible in most countries, except for public occupational therapy in the United Kingdom. Access to public services is higher than to private services in all countries. Balneotherapy, private and public, and specialist nurses, private and public, have limited availability. Private sports medicine access is higher than public sports medicine.

### Rheumatology and highly specialised services

#### Access to rheumatology

Table 5 shows how difficult it is to get a referral to rheumatology in case of MSK complaints. The countries where citizens perceive getting a referral to rheumatology as most difficult are Spain, the UK, and Germany. GPs perceive the most difficulty in referring to rheumatology in Germany and the least in the UK. Rheumatologists also note the perceived difficulty in Germany. Greek rheumatologists considered the referral to rheumatology a straightforward referral.

**Table 5:** Difficulty in referring a patient with MSK complaints to rheumatology in 7 European countries

**Difficulty in getting a referral**	DE	HE	HU	NE	ES	SE	UK	Total
% responses of users								
Very difficult	13		4		11	7	11	7
Difficult	13	8	13	8	22	10	17	14
Neither easy nor difficult	19	33	30	25	33	33	28	29
Easy	25	42	22	25	17	10	6	19
Very easy			4			3	6	2
Difficulty score*	219	217	213	158	278	197	222	
% responses of GPs								
Very difficult	20							3
Difficult	60	29						16
Neither easy nor difficult		43		57	57	75		44
Easy	20	29	100	43	29			28
Very easy					14	25	100	9
Difficulty score*	380	300	200	257	243	250	100	
% responses of rheumatologists								
Very difficult	15				5			5
Difficult	15				16		17	10
Neither easy nor difficult	31		50		32	71	50	35
Easy	31	50	50	100	32	29	17	38
Very easy	8	50			16		17	13
Difficulty score*	300	150	250	200	263	271	267	

The cells represent the percentage (%) per difficulty level per country. They do not add up to 100% as “I don’t know” was removed from the count and the tables

*A “Difficulty score” was calculated as a weighted sum of scores, with “*very difficult*” responses multiplied by 5 and “*very easy*” by 1, with all other scores in between (See Methods)

Abbreviations: DE, Germany; HE, Greece; HU, Hungary; NE, Netherlands; ES, Spain; SE, Sweden; UK, United Kingdom; GPs, general practitioners

#### Access to highly specialised services

The country with the highest access to all specialised units is the UK, and the countries with the lowest perceived access are Greece and Hungary. Early arthritis clinics, and to a lesser extent, early SpA clinics, were moderately to highly available in all countries despite the responses of GPs being discordant with those of rheumatologists (Supplementary File; Table S4).

#### Diagnostic delay and need for triage

Table 6 represents the percentage of responses to the average time needed to get a rheumatological diagnosis per country based on the survey respondents’ experience. The country where the users felt the time to a diagnosis was the longest was Spain, and the shortest was Sweden. For the GPs, Germany was the longest, and Greece and Sweden were the shortest. For the rheumatologists, the shortest was the Netherlands, and the longest were Germany and the UK.

Rheumatologists were asked whether triaging the patients referred to rheumatology is common practice in their settings (Table 6). Triage is commonplace in the Netherlands, UK, and Sweden and is frequent in Germany, but it is not always present in rheumatology settings in Spain, Hungary, or Greece.

**Table 6:** Time to get a rheumatological diagnosis in 7 European countries, by participants’ perception and country

Time to rheumatological diagnosis	DE	HE	HU	NE	ES	SE	UK
% **users’ responses**							
Less than 1 month		17	17	9			11
1–3 months		25	11	18	6	10	11
4–6 months	13		22		11	7	6
7–12 months	7	17	6	18	11	3	
More than 12 months	20	8	17	18	33	10	28
Experienced delay score*	167	175	211	209	256	107	189
**% GPs responses**							
Less than 1 month	0	0	0	0	0	0	0
1–3 months	0	43	0	43	29	50	100
4–6 months	40	0	100	29	43	25	0
7–12 months	60	14	0	14	29	0	0
More than 12 months	0	0	0	0	0	0	0
Experienced delay score*	360	143	300	229	300	175	200
**% rheumatologists’ responses**							
Less than 1 month	0	17	0	40	5	0	0
1–3 months	0	17	0	20	26	29	17
4–6 months	15	33	38	20	21	71	0
7–12 months	85	33	63	20	37	0	67
More than 12 months	0	0	0	0	11	0	17
Experienced delay score*	385	283	363	220	321	271	383
**Triage in place in rheumatology (%)**	75	17	38	100	53	100	100

The cells represent a percentage per experienced length per country. They do not add up to 100% as “I did not get a diagnosis” was removed from the count

Abbreviations: DE, Germany; HE, Greece; HU, Hungary; NE, Netherlands; ES, Spain; SE, Sweden; UK, United Kingdom; GPs, general practitioners

*An “**experienced delay score**” was calculated as a weighted sum of scores being “*More than 12 months*” responses multiplied by 5 and “*Less than 1 month*” by 1, with all other scores in between (The options “*I don’t have a diagnosis*” or “*I don’t know*” were removed) (See Methods)

#### Patients followed up in rheumatology

Rheumatologists responded on the type of patients generally followed up in rheumatology clinics in their countries (Supplementary File; TableS5). There was a significant variation across countries. Patients with OA, gout, or soft-tissue pain were rarely seen in rheumatology in Sweden, the UK, and Germany, some more in Hungary and often in Greece, the Netherlands, and Spain. In all countries, those with IRDs were more commonly followed up in rheumatology. In most instances, all other types of patients are followed until an IRD diagnosis is ruled out or the disease is moderately controlled.

#### Organisational aspects

Clinicians were asked how organised their healthcare systems were (Supplementary File; Table S6). The countries where the respondents felt best organised were Sweden, the Netherlands, and the UK; the poorest were Greece and Hungary.

They were also asked about the possibility of direct communication between care levels (not just letters or via the patient) (Supplementary File; Table S7). According to the rheumatologists, access was only relevant in Sweden (moderate in Spain and the UK). Still, the GPs responded more frequently that they had access to direct communication with the specialist. Per their comments, one-way communication is possible in all countries (e.g., in Hungary, the patient notes from specialist care are available to the GPs in a national cloud system of healthcare records, or the Netherlands, electronic letters or personal emails are frequent). Spain has developed an e-consultation system, but it is not yet implemented nationwide. In the UK, the GP can communicate with the rheumatologist via an ‘advice and guidance’ service, which the GP must initiate.

## Discussion

In this survey, we have identified some differences across seven European countries on the healthcare systems characteristics and touchpoints once a person starts with MSK symptoms. Notably, countries did not differ in help-seeking behaviours of citizens but in the pathways to rheumatology, with varying levels of access to specialists and other resources, highlighting the different pathways to diagnosis, assessment, and referral to healthcare professionals and rheumatology, depending on the country.

Interestingly, we observed variability within and between the responses from citizens, with and without RMDs, GPs, and rheumatologists. One might think that the health systems and access to resources are specified and are familiar to all stakeholders within the country. This is the reasoning behind asking a single informant or department, for instance, at the country level or obtaining the information from the ministry of health, as in OCDE or European Commission reports [29, 30]. However, this is not the reality or not how the users experience it [31–33]. They may reflect the users’ wrong perception of access, either because of preconceptions or past experiences of being denied access in similar circumstances. Accessing some resources might be so unclear that they might look blocked or unavailable when, in reality, the resource is available, or was not accessible in the past, or it changed but was not adequately communicated. This often occurs due to a shortage of healthcare professionals [34, 35]. The variability in the responses between GPs and rheumatologists—the former being less optimistic about access to rheumatological tests than the latter— also reflects the lack of awareness of access between healthcare levels. It reflects a complex reality of miscommunication, also revealed in the questions on organisational aspects, that ultimately impacts the experience of the users, both patients and professionals [27, 36].

Internet searches (47–59%) and GP visits (60–76%) were the most common initial help-seeking behaviours for individuals with MSK symptoms. Internet searches for health information influence traditional healthcare touchpoints, such as GP consultations, in complex ways. While the Internet is not a formal touchpoint in the healthcare system, it significantly influences patient behaviour and expectations during GP visits, as shown in a survey of Polish adults, where 45% searched the Internet to decide whether to go to the GP [37]. Likewise, a Belgian study showed that 60% of those who searched the Internet for symptoms were reassured, and one-third were worried after the search [38]. Internet-derived information during consultations can enhance communication between patients and GPs, fostering a better understanding of symptoms and diagnoses [38] and more informed discussions during consultations [39]. There is also a correlation with healthcare access. Increased internet searches for walk-in clinics correlate with better access to primary care, suggesting that patients are actively seeking alternatives when traditional access is limited [40]. This trend indicates that while the internet is not a direct touchpoint, it plays a crucial role in shaping patient pathways to care. However, reliance on internet searches may also contribute to misinformation, creating anxiety or distrust in the healthcare system [41]. This highlights the need for effective communication strategies between patients and healthcare providers to mitigate the potential negative impacts of online health information. Therefore, any tool to facilitate diagnosis suspicion, like the ones the SPIDeRR consortium is designing [42], should consider this continuum of actions.

Primary care is the most common first touchpoint at the healthcare system level for people with MSK complaints. The number of consultations is large as it is its variability of symptoms [43]. The GP is a gatekeeper for the system in most settings, although in some countries, like Greece, directly visiting a rheumatologist for MSK complaints is possible. GPs can manage prevalent RMDs, such as OA, FM, or gout [44–46] but the differential diagnosis of MSK complaints and their treatment is challenging without proper training on RMDs. In this sense, in the survey, GPs’ knowledge of RMDs was generally perceived as moderate at the most. Several studies have shown that GPs often lack confidence in assessing IRDs [47, 48]. Any strategy aimed at improving the identification and adequate management of RMDs in primary care would need to increase GPs’ confidence, either through training or support systems [24].

GPs can refer to rheumatology or other specialists related to MSK health, like a physiotherapist or an orthopaedic surgeon. These latter can also be accessed directly by the patient in some health systems. Ideally, patients with a suspicion of IRD should be referred to a rheumatologist to avoid delay of a proper diagnosis and treatment and, thus, improve their outcome [49]. However, symptoms alone are often insufficient for diagnosis, requiring additional testing. Access to rheumatological tests, medications, and non-rheumatology healthcare professionals varies significantly between countries, or at least the perception of accessibility. Swedish respondents opine access to tests is high, while Hungarians perceive limited access. Commonly available diagnostic tests, like anti-CCP or ACPA, and joint symptom assessments can effectively distinguish between patients who need a rheumatology referral and those who do not [50]. The most determinant factor limiting referral is the shortage of rheumatology services and long waiting times [36, 47, 49–51]. Swedish respondents opine diagnosis times are sufficiently short, while Hungarians perceive long diagnosis times. These perceptions should be contrasted with the availability of rheumatologists in their countries. Access to rheumatological pharmacological and non-pharmacological treatment in primary care could maintain the patients in good condition until an appointment in rheumatology is possible [50]. However, some rheumatologists complain that these increase diagnostic delays [49, 50].

The next touchpoint of people with MSK complaints is the rheumatology referral, sometimes to a highly specialised unit, such as an early arthritis clinic. Once in rheumatology, the patient can be returned to primary care, with or without a visit (depending on triage protocols), stay and be followed up by rheumatology, entirely or in shared care with the GP, depending on the diagnosis. Some organisational aspects are relevant to this pathway, such as the availability of specialised units or pathways present in a few countries or the communications between healthcare providers and levels, with certainly much room for improvement.

This study has several limitations, mainly because it is a survey and not an audit study. We had previously researched information about the healthcare systems in the target countries [36, 37] and could not find detailed information about rheumatology. Therefore, we envisioned a survey of different stakeholders as an efficient way to understand the context better. A strength of the study is precisely the diverse geographic representation and inclusion of different professional perspectives. Another limitation is a potential selection bias with the snowball technique; however, we specifically asked for representative participants. Their description confirms that they represent an adequate spectrum of potential users of the seven health systems. Finally, we could not avoid unequal sample sizes across groups due to the difficulty of recruitment, especially GPs.

This study identifies previously undocumented patterns in RMD patient pathways. Notably, the heavy reliance on internet searches as an initial step in healthcare-seeking behavior has not been highlighted in prior research. Furthermore, the substantial variation in access to sacroiliac MRI between Hungary and Spain compared to the UK’s early arthritis clinics demonstrates unique regional disparities. The study also reveals that despite the availability of glucocorticoids across all countries, GPs’ limited knowledge of RMDs remains a critical barrier to timely diagnosis and treatment. These insights contribute valuable new evidence to the field and can inform future policy interventions aimed at standardizing diagnostic pathways across Europe.

In conclusion, these findings highlight significant variations in healthcare pathways, access to services, and perceptions across European countries for patients with MSK complaints. The results emphasise the importance of the Internet and primary care as initial touchpoints while revealing discrepancies in how different actors view the patient journey in different countries. We will use this information as context for implementing the tools developed in the SPIDeRR project.

## Electronic supplementary material

Below is the link to the electronic supplementary material.

Supplementary Material 1

Supplementary Material 2

Supplementary Material 3

Supplementary Material 4

Supplementary Material 5

## Data Availability

The datasets used and/or analyzed during the current study are fully anonymized and available from the corresponding author on reasonable request. All data relevant to the study are included in the article or uploaded as supplementary material. For further questions regarding the reuse of data, please contact the corresponding authors.

## Declarations

### Generative AI and AI-assisted technologies in the writing process

During the preparation of this work the authors used OpenAI ChatGPT (Version 4) in order to improve grammar, language and readability. After using this tool/service, the authors reviewed and edited the content as needed and take full responsibility for the content of the publication.

## References

[1] Lai KN et al (2016) IgA nephropathy. Nat Rev Dis Primers 2:16001 27189177 10.1038/nrdp.2016.1

[2] Simpson C et al (2005) The patient’s journey: rheumatoid arthritis. BMJ 331(7521):887–889 16223822 10.1136/bmj.331.7521.887 PMC1255800

[3] Burmester GR (2015) Rheumatische und Muskuloskelettale Erkrankungen– eine wissenschaftliche und klinische herausforderung. Drug Res (Stuttg) 65:01 10.1055/s-0035-1558059 26536183

[4] Beard JR et al (2016) The world report on ageing and health: a policy framework for healthy ageing. Lancet 387(10033):2145–2154 26520231 10.1016/S0140-6736(15)00516-4 PMC4848186

[5] Koch-Institut R (2024) *Themenschwerpunkt: Muskuloskelettale Erkrankungen*. [cited 2024 30.07.2024]; Available from: https://www.rki.de/DE/Content/Gesundheitsmonitoring/Themen/Chronische_Erkrankungen/Muskel_Skelett_System/Muskel_Skelett_System_node.html

[6] Firestein GS, McInnes IB (2017) Immunopathogenesis Rheumatoid Arthritis Immun 46(2):183–196 10.1016/j.immuni.2017.02.006 PMC5385708 28228278

[7] Loza E et al (2008) Burden of disease across chronic diseases: a health survey that measured prevalence, function, and quality of life. J Rheumatol 35(1):159–165 17937459

[8] Choy E et al (2010) A patient survey of the impact of fibromyalgia and the journey to diagnosis. BMC Health Serv Res 10:102 20420681 10.1186/1472-6963-10-102 PMC2874550

[9] Carmona L et al (2001) The burden of musculoskeletal diseases in the general population of Spain: results from a National survey. Ann Rheum Dis 60(11):1040–1045 11602475 10.1136/ard.60.11.1040 PMC1753418

[10] Schett G, Gravallese E (2012) Bone erosion in rheumatoid arthritis: mechanisms, diagnosis and treatment. Nat Rev Rheumatol 8(11):656–664 23007741 10.1038/nrrheum.2012.153 PMC4096779

[11] van Nies JA et al (2014) What is the evidence for the presence of a therapeutic window of opportunity in rheumatoid arthritis? A systematic literature review. Ann Rheum Dis 73(5):861–870 23572339 10.1136/annrheumdis-2012-203130

[12] Nell VP et al (2004) Benefit of very early referral and very early therapy with disease-modifying anti-rheumatic drugs in patients with early rheumatoid arthritis. Rheumatology (Oxford) 43(7):906–914 15113999 10.1093/rheumatology/keh199

[13] Danve A, Deodhar A (2019) Axial spondyloarthritis in the USA: diagnostic challenges and missed opportunities. Clin Rheumatol 38(3):625–634 30588555 10.1007/s10067-018-4397-3

[14] Zhao SS et al (2021) Diagnostic delay in axial spondyloarthritis: a systematic review and meta-analysis. Rheumatology (Oxford) 60(4):1620–1628 33428758 10.1093/rheumatology/keaa807

[15] Lubrano E, De Socio A, Perrotta FM (2018) Unmet needs in axial spondyloarthritis. Clin Rev Allergy Immunol 55(3):332–339 28779298 10.1007/s12016-017-8637-0

[16] Barhamain AS et al (2017) The journey of rheumatoid arthritis patients: a review of reported lag times from the onset of symptoms. Open Access Rheumatol 9:139–150 28814904 10.2147/OARRR.S138830 PMC5546831

[17] Jovaní V et al (2017) Understanding how the diagnostic delay of spondyloarthritis differs between women and men: A systematic review and metaanalysis. J Rheumatol 44(2):174–183 27980009 10.3899/jrheum.160825

[18] Sykes MP et al (2015) Delay to diagnosis in axial spondyloarthritis: are we improving in the UK? Rheumatology 54(12):2283–2284 26283680 10.1093/rheumatology/kev288

[19] Doebl S et al (2022) Comparing the impact of symptoms and health care experiences of people who have and have not received a diagnosis of fibromyalgia: A Cross-Sectional survey within the PACFiND study. Arthritis Care Res (Hoboken) 74(11):1894–1902 34085414 10.1002/acr.24723

[20] Xiang L et al (2021) Interval between symptom onset and diagnosis among patients with autoimmune rheumatic diseases in a multi-ethnic Asian population. Int J Rheum Dis 24(8):1061–1070 34232556 10.1111/1756-185X.14165

[21] Fallahi S, Jamshidi AR (2016) Diagnostic delay in ankylosing spondylitis: related factors and prognostic outcomes. Arch Rheumatol 31(1):24–30 29900990 10.5606/ArchRheumatol.2016.5562 PMC5827863

[22] Möttönen T et al (2002) Delay to institution of therapy and induction of remission using single-drug or combination-disease-modifying antirheumatic drug therapy in early rheumatoid arthritis. Arthritis Rheum 46(4):894–898 11953964 10.1002/art.10135

[23] Chan KW et al (1994) The lag time between onset of symptoms and diagnosis of rheumatoid arthritis. Arthritis Rheum 37(6):814–820 8003053 10.1002/art.1780370606

[24] Woolf AD (2002) Specialist training in rheumatology in Europe. Rheumatology (Oxford) 41(9):1062–1066 12209042 10.1093/rheumatology/41.9.1062

[25] SPIDeRR consortium, Stratification of Patients using advanced Integrative modeling of Data Routinely acquired for diagnosing Rheumatic complaints (SPIDeRR) (2023) Horizon Europe (HORIZON): https://cordis.europa.eu/project/id/101080711

[26] Zimba O, Gasparyan AY (2023) Designing, conducting, and reporting survey studies: A primer for researchers. J Korean Med Sci 38(48):e403 38084027 10.3346/jkms.2023.38.e403 PMC10713437

[27] Oton T, Villalobos M, Carmona L (2024) The patient journey of patients with rheumatic diseases: A systematic review. Submitted)

[28] Neuberger J (1999) Do we need a new word for patients? Lets do away with patients. BMJ 318(7200):1756–1757 10381717 10.1136/bmj.318.7200.1756 PMC1116090

[29] OECD (2023) *Health System Characteristics Survey*

[30] Commission E (2023) *State of Health in the EU: Synthesis Report 2023*.: Luxembourg: Publications Office of the European Union, 2023

[31] Putrik P et al (2014) Variations in criteria regulating treatment with reimbursed biologic DMARDs across European countries. Are differences related to country’s wealth? Ann Rheum Dis 73(11):2010–2021 23940213 10.1136/annrheumdis-2013-203819

[32] Jovani V et al (2012) Variability in resource consumption in patients with spondyloarthritis in Spain. Preliminary descriptive data from the emar II study. Reumatol Clin 8(3):114–119 22503152 10.1016/j.reuma.2012.01.011

[33] López-Longo FJ et al (2018) Variability in the prescription of biological drugs in rheumatoid arthritis in Spain: a multilevel analysis. Rheumatol Int 38(4):589–598 29368023 10.1007/s00296-018-3933-4

[34] van den Broek-Altenburg E et al (2021) Understanding the factors that affect the appropriateness of rheumatology referrals. BMC Health Serv Res 21(1):1124 34666756 10.1186/s12913-021-07036-5 PMC8527790

[35] Braun J et al (2024) [Rheumatological care in Germany: memorandum of the German society for rheumatology and clinical immunology 2024]. Z Rheumatol 83(Suppl 2):249–284 39136764 10.1007/s00393-024-01539-2

[36] Villaverde V et al (2011) [Characteristics of early arthritis units that May be associated with better referral efficiency: survey of SERAP units]. Reumatol Clin 7(4):236–240 21794824 10.1016/j.reuma.2010.11.014

[37] Bujnowska-Fedak MM, Węgierek P (2020) The impact of online health information on patient health behaviours and making decisions concerning health. Int J Environ Res Public Health, 17(3) 10.3390/ijerph17030880 PMC7037991 32023828

[38] Van Riel N et al (2017) The effect of Dr Google on doctor–patient encounters in primary care: a quantitative, observational, cross-sectional study. BJGP Open, 1(2): p. bjgpopen17X100833. 10.3399/bjgpopen17X100833 PMC6169945 30564661

[39] Seguin M et al (2018) Protocol paper for the ‘Harnessing resources from the internet to maximise outcomes from GP consultations (HaRI)’ study: a mixed qualitative methods study. BMJ Open 8(8):e024188 30099404 10.1136/bmjopen-2018-024188 PMC6089307

[40] Ssendikaddiwa J, Lavergne R (2019) Access to primary care and internet searches for Walk-In clinics and emergency departments in Canada: observational study using Google trends and population health survey data. JMIR Public Health Surveill 5(4):e13130 31738175 10.2196/13130 PMC6913775

[41] Krusche M et al (2020) [Mobile apps and their usage in rheumatology]. Z Rheumatol 79(6):554–561 32472178 10.1007/s00393-020-00822-2

[42] Lundberg K et al (2023) Population-based user-perceived experience of rheumatic?? A novel digital symptom-checker in rheumatology. RMD Open 9(2):e002974 37094982 10.1136/rmdopen-2022-002974 PMC10152040

[43] Haas R et al (2023) Prevalence and characteristics of musculoskeletal complaints in primary care: an analysis from the population level and analysis reporting (POLAR) database. BMC Prim Care 24(1):40 36739379 10.1186/s12875-023-01976-z PMC9898983

[44] Sidari A, Hill E (2018) Diagnosis and treatment of gout and pseudogout for everyday practice. Prim Care 45(2):213–236 29759121 10.1016/j.pop.2018.02.004

[45] Byrne A et al (2023) Patient and primary care practitioners’ perspectives on consultations for fibromyalgia: a qualitative evidence synthesis. Prim Health Care Res Dev 24:e58 37750736 10.1017/S1463423623000506 PMC10540196

[46] Littlejohn EA, Monrad SU (2018) Early diagnosis and treatment of rheumatoid arthritis. Prim Care 45(2):237–255 29759122 10.1016/j.pop.2018.02.010

[47] Meyfroidt S et al (2015) A general practice perspective on early rheumatoid arthritis management: A qualitative study from Flanders. Eur J Gen Pract 21(4):231–237 26679974 10.3109/13814788.2015.1084279

[48] Adizie T et al (2018) Knowledge of features of inflammatory back pain in primary care in the West Midlands: a cross-sectional survey in the united Kingdom. Rheumatol Int 38(10):1859–1863 30027350 10.1007/s00296-018-4058-5

[49] Foster NE, Hartvigsen J, Croft PR (2012) Taking responsibility for the early assessment and treatment of patients with musculoskeletal pain: a review and critical analysis. Arthritis Res Ther 14(1):205 22404958 10.1186/ar3743 PMC3392833

[50] Garcia-Montoya L et al (2022) Prioritising referrals of individuals at-risk of RA: guidance based on results of a 10-year National primary care observational study. Arthritis Res Ther 24(1):26 35042555 10.1186/s13075-022-02717-w PMC8767684

[51] Juanola Roura X et al (2015) Reccomendations for the detection, study and referral of inflammatory low-back pain in primary care. Reumatol Clin 11(2):90–98 25241260 10.1016/j.reuma.2014.04.007

