# Supplementary material for: The journey of patients with musculoskeletal complaints in Europe: a cross-sectional European survey
Source: Rheumatol Int. 2025 Apr 18;45(5):107. doi: 10.1007/s00296-025-05863-x (PMC12008060; doi:10.1007/s00296-025-05863-x)
Supplement: Supplementary file 1 — Supplementary Material 1 [file 296_2025_5863_MOESM1_ESM.docx]

The Journey of Patients with Musculoskeletal Complaints in Europe: A Pan-European Survey among Citizens and Health Care Professionals

Supplementary Materials

[Table S1. Details of the survey participants. 1](#_Toc175127933)

[Table S2. Level of knowledge about RMDs by GPs, as perceived by themselves and rheumatologists, per country. 3](#_Toc175127934)

[Table S3. Perceived access to specific professionals related to MSK Health based on country and stakeholder perception. 4](#_Toc175127935)

[Table S4. Access to specialised care units in rheumatology or health pathways established for RMDs. 6](#_Toc175127936)

[Table S5. Type of patients commonly seen in rheumatology. 7](#_Toc175127937)

[Table S6. Level of perceived organisation of the health system. 7](#_Toc175127938)

[Table S7. Direct communication between healthcare levels. 7](#_Toc175127939)

# Table S1. Details of the survey participants.

| **Descriptor** | **n** | **%** |
| --- | --- | --- |
| **Citizens** | | |
| Rheumatic disease |  |  |
| No | 69 | 48.9 |
| No, but symptoms | 8 | 5.7 |
| Yes, diagnosed with an RMD | 64 | 45.4 |
| Osteoarthritis | 10 |  |
| Rheumatoid arthritis | 9 |  |
| Axial spondyloarthritis | 5 |  |
| Psoriatic arthritis | 5 |  |
| Arthritis | 2 |  |
| Fibromyalgia | 2 |  |
| Gout | 1 |  |
| Juvenile idiopathic arthritis | 1 |  |
| Osteoporosis | 1 |  |
| Seronegative polyarthritis | 1 |  |
| Peripheral spondyloarthritis | 1 |  |
| Undifferentiated connective tissue disease | 1 |  |
| Undifferentiated spondyloarthritis | 1 |  |
| Scleroderma, vasculitis, Sjögren syndrome | 1 |  |
| Age |  |  |
| Less than 30 | 20 | 14.2 |
| 30 to 50 | 63 | 44.7 |
| More than 50 | 58 | 41.1 |
| Gender |  |  |
| Female | 91 | 64.5 |
| Male | 50 | 35.5 |
| Socioeconomic level |  |  |
| Very high | 6 | 4.3 |
| High | 61 | 43.6 |
| Medium | 67 | 47.9 |
| Low | 6 | 4.3 |
| **Rheumatologists** | | |
| Sector |  |  |
| Public system | 60 | 89.6 |
| Private system | 5 | 7.5 |
| Both | 1 | 1.5 |
| Retired | 1 | 1.5 |
| Type of practice |  |  |
| In a group/centre, sharing the pool of patients | 47 | 70.2 |
| In a group/centre, not sharing the pool | 12 | 17.9 |
| Alone (solo practice) | 4 | 6.0 |
| I do not attend to patients currently | 2 | 3.0 |
| Other* | 2 | 3.0 |
| Gender |  |  |
| Female | 32 | 47.8 |
| Male | 35 | 52.2 |
| Time practising |  |  |
| Less than 5 years | 7 | 10.4 |
| 5 to 20 years | 36 | 53.7 |
| More than 20 years | 24 | 35.8 |
| **GPs** | | |
| Sector |  |  |
| Public system | 36 | 94.7 |
| Private system | 1 | 2.6 |
| Other** | 1 | 2.6 |
| Setting |  |  |
| Urban | 30 | 79.0 |
| Rural | 6 | 18.4 |
| Other^†^ | 1 | 2.6 |
| Gender |  |  |
| Female | 26 | 68.4 |
| Male | 12 | 31.6 |
| Time practising |  |  |
| Less than 5 years | 14 | 38.9 |
| 5 to 20 years | 12 | 33.3 |
| More than 20 years | 10 | 27.8 |
| Type of practice |  |  |
| In a group/centre, sharing the pool of patients | 7 | 18.4 |
| In a group/centre, not sharing the pool | 24 | 63.2 |
| Alone (solo practice) | 3 | 7.9 |
| I do not attend to patients currently | 2 | 5.3 |
| Other^‡^ | 2 | 5.3 |

*1 Director of Health area and 1 faculty at a university

** Global company.

†Both.

‡ In a centre, sharing patients with one other doctor and not sharing patients with five others (Netherlands). Group/centre with a pool of patients for the centre (nurses, etc) but individual physicians for each patient (Sweden).

# Table S2. Level of knowledge about RMDs by GPs, as perceived by themselves and rheumatologists, per country.

|  | **DE** | **HE** | **HU** | **NE** | **ES** | **SE** | **UK** | **Total** |
| --- | --- | --- | --- | --- | --- | --- | --- | --- |
| **Rheumatologists' perspective** |  |  |  |  |  |  |  |  |
| Very knowledgeable | 0 | 0 | 0 | 0 | 0 | 14 | 0 | 2 |
| Knowledgeable | 15 | 17 | 0 | 0 | 21 | 71 | 17 | 20 |
| Moderately knowledgeable | 62 | 67 | 50 | 80 | 63 | 14 | 67 | 58 |
| Not very knowledgeable | 23 | 17 | 38 | 20 | 16 | 0 | 17 | 19 |
| Not knowledgeable at all | 0 | 0 | 13 | 0 | 0 | 0 | 0 | 2 |
| Knowledge score | 292 | 300 | 238 | 280 | 305 | 400 | 300 |  |
| **GPs' perspective** |  |  |  |  |  |  |  |  |
| Very knowledgeable | 0 | 0 | 0 | 0 | 0 | 25 | 0 | 3 |
| Knowledgeable | 20 | 0 | 0 | 14 | 43 | 50 | 0 | 22 |
| Moderately knowledgeable | 80 | 43 | 0 | 86 | 57 | 25 | 100 | 59 |
| Not very knowledgeable | 0 | 57 | 100 | 0 | 0 | 0 | 0 | 16 |
| Not knowledgeable at all | 0 | 0 | 0 | 0 | 0 | 0 | 0 | 0 |
| Knowledge score | 320 | 243 | 200 | 314 | 343 | 400 | 300 |  |

Cells represent the percentage responding affirmatively per country.

# Table S3. Perceived access to specific professionals related to MSK Health based on country and stakeholder perception.

| **Service** | **Stakeholder perspective** | **DE** | **HE** | **HU** | **NE** | **ES** | **SE** | **UK** | **Total** |
| --- | --- | --- | --- | --- | --- | --- | --- | --- | --- |
| Primary care doctors – Public | Patients & the public | 100 | 67 | 100 | 92 | 100 | 97 | 100 | 95 |
|  | Rheumatologists | 100 | 100 | 100 | 80 | 100 | 100 | 100 | 98 |
|  | GPs | 100 | 100 | 100 | 100 | 100 | 100 | 100 | 100 |
|  |  |  |  |  |  |  |  |  |  |
| Primary care doctors – Private | Patients & the public | 91 | 89 | 44 | 67 | 79 | 90 | 100 | 76 |
|  | Rheumatologists | 100 | 80 | 50 | 50 | 86 | 100 | 50 | 79 |
|  | GPs | 100 | 100 | 0 | 40 | 83 | 100 | 100 | 81 |
|  |  |  |  |  |  |  |  |  |  |
| Orthopaedics – Public | Patients & the public | 94 | 100 | 95 | 91 | 83 | 79 | 88 | 89 |
|  | Rheumatologists | 100 | 100 | 100 | 80 | 100 | 100 | 100 | 98 |
|  | GPs | 100 | 100 | 100 | 100 | 100 | 100 | 100 | 100 |
|  |  |  |  |  |  |  |  |  |  |
| Orthopaedics – Private | Patients & the public | 91 | 89 | 78 | 70 | 86 | 30 | 56 | 73 |
|  | Rheumatologists | 100 | 100 | 100 | 75 | 100 | 100 | 100 | 98 |
|  | GPs | 100 | 100 | 100 | 100 | 100 | 100 | 100 | 100 |
|  |  |  |  |  |  |  |  |  |  |
| Rheumatology – Public | Patients & the public | 94 | 100 | 100 | 92 | 88 | 79 | 94 | 91 |
|  | Rheumatologists | 100 | 100 | 100 | 80 | 100 | 100 | 100 | 98 |
|  | GPs | 100 | 86 | 100 | 100 | 100 | 100 | 100 | 97 |
|  |  |  |  |  |  |  |  |  |  |
| Rheumatology – Private | Patients & the public | 91 | 100 | 82 | 22 | 85 | 40 | 44 | 69 |
|  | Rheumatologists | 100 | 100 | 100 | 50 | 100 | 100 | 100 | 96 |
|  | GPs | 100 | 100 | 100 | 60 | 100 | 50 | 100 | 84 |
|  |  |  |  |  |  |  |  |  |  |
| Physiotherapy – Public | Patients & the public | 94 | 55 | 95 | 92 | 33 | 90 | 94 | 82 |
|  | Rheumatologists | 100 | 100 | 100 | 80 | 89 | 100 | 100 | 95 |
|  | GPs | 100 | 71 | 100 | 86 | 100 | 100 | 100 | 91 |
|  |  |  |  |  |  |  |  |  |  |
| Physiotherapy – Private | Patients & the public | 100 | 100 | 82 | 60 | 93 | 70 | 82 | 84 |
|  | Rheumatologists | 100 | 100 | 100 | 75 | 100 | 100 | 100 | 98 |
|  | GPs | 100 | 100 | 100 | 100 | 100 | 75 | 100 | 96 |
|  |  |  |  |  |  |  |  |  |  |
| Occupational therapy – Public | Patients & the public | 44 | 27 | 38 | 58 | 7 | 68 | 82 | 50 |
|  | Rheumatologists | 92 | 50 | 71 | 80 | 63 | 100 | 100 | 77 |
|  | GPs | 100 | 14 | 0 | 71 | 43 | 75 | 100 | 55 |
|  |  |  |  |  |  |  |  |  |  |
| Occupational therapy – Private | Patients & the public | 36 | 44 | 53 | 50 | 54 | 60 | 50 | 50 |
|  | Rheumatologists | 92 | 60 | 50 | 50 | 80 | 80 | 83 | 75 |
|  | GPs | 100 | 17 | 0 | 40 | 40 | 75 | 100 | 46 |
|  |  |  |  |  |  |  |  |  |  |
| Rehabilitation – Public | Patients & the public | 88 | 27 | 76 | 75 | 75 | 86 | 69 | 74 |
|  | Rheumatologists | 100 | 83 | 100 | 80 | 100 | 100 | 100 | 95 |
|  | GPs | 100 | 29 | 100 | 100 | 100 | 100 | 100 | 84 |
|  |  |  |  |  |  |  |  |  |  |
| Rehabilitation – Private | Patients & the public | 82 | 78 | 65 | 60 | 85 | 60 | 44 | 68 |
|  | Rheumatologists | 100 | 60 | 67 | 50 | 100 | 60 | 83 | 83 |
|  | GPs | 100 | 67 | 0 | 40 | 100 | 75 | 0 | 68 |
|  |  |  |  |  |  |  |  |  |  |
| Balneotherapy – Public | Patients & the public | 25 | 9 | 67 | 8 | 14 | 7 | 7 | 21 |
|  | Rheumatologists | 83 | 17 | 100 | 20 | 0 | 29 | 50 | 39 |
|  | GPs | 50 | 0 | 100 | 0 | 0 | 0 | 0 | 10 |
|  |  |  |  |  |  |  |  |  |  |
| Balneotherapy – Private | Patients & the public | 18 | 22 | 47 | 30 | 57 | 10 | 25 | 33 |
|  | Rheumatologists | 85 | 25 | 100 | 25 | 73 | 20 | 33 | 62 |
|  | GPs | 50 | 33 | 0 | 0 | 60 | 0 | 100 | 29 |
|  |  |  |  |  |  |  |  |  |  |
| Specialist nurses – Public | Patients & the public | 19 | 27 | 14 | 42 | 47 | 62 | 88 | 44 |
|  | Rheumatologists | 67 | 33 | 86 | 80 | 84 | 71 | 100 | 76 |
|  | GPs | 50 | 0 | 0 | 100 | 29 | 75 | 100 | 48 |
|  |  |  |  |  |  |  |  |  |  |
| Specialist nurses – Private | Patients & the public | 40 | 44 | 41 | 20 | 42 | 40 | 33 | 38 |
|  | Rheumatologists | 69 | 25 | 50 | 50 | 29 | 40 | 50 | 46 |
|  | GPs | 100 | 33 | 0 | 20 | 0 | 50 | 0 | 29 |
|  |  |  |  |  |  |  |  |  |  |
| Psychologists (specialists in pain) – Public | Patients & the public | 69 | 36 | 24 | 64 | 27 | 54 | 56 | 47 |
|  | Rheumatologists | 83 | 67 | 86 | 75 | 47 | 43 | 83 | 66 |
|  | GPs | 100 | 14 | 0 | 86 | 29 | 100 | 100 | 59 |
|  |  |  |  |  |  |  |  |  |  |
| Psychologists (specialists in pain) – Private | Patients & the public | 73 | 67 | 71 | 60 | 62 | 60 | 60 | 65 |
|  | Rheumatologists | 85 | 100 | 50 | 50 | 53 | 60 | 100 | 70 |
|  | GPs | 100 | 33 | 0 | 80 | 40 | 25 | 100 | 52 |
|  |  |  |  |  |  |  |  |  |  |
| Sports doctors – Public | Patients & the public | 69 | 45 | 71 | 75 | 13 | 41 | 25 | 48 |
|  | Rheumatologists | 92 | 67 | 86 | 80 | 17 | 71 | 80 | 62 |
|  | GPs | 100 | 29 | 100 | 100 | 0 | 50 | 0 | 52 |
|  |  |  |  |  |  |  |  |  |  |
| Sports doctors - Private | Patients & the public | 73 | 78 | 76 | 60 | 71 | 70 | 58 | 70 |
|  | Rheumatologists | 92 | 100 | 83 | 50 | 93 | 60 | 100 | 87 |
|  | GPs | 100 | 67 | 0 | 100 | 83 | 100 | 100 | 85 |

Cells represent the percentage of stakeholders answering “Yes” per country.

# Table S4. Access to specialised care units in rheumatology or health pathways established for RMDs.

|  | | **DE** | **HE** | **HU** | **NE** | **ES** | **SE** | **UK** | **Total** |
| --- | --- | --- | --- | --- | --- | --- | --- | --- | --- |
| **Rheumatologists** | |  |  |  |  |  |  |  |  |
| Specialised care units | | 50 | 67 | 63 | 100 | 67 | 100 | 100 | 72 |
| RA (early arthritis clinics, etc.) | | 50 | 50 | 63 | 80 | 67 | 100 | 100 | 69 |
| SpA (early SpA clinics, etc.) | | 43 | 50 | 50 | 20 | 43 | 86 | 100 | 52 |
| Osteoporosis (FLS, etc.) | | 0 | 33 | 38 | 60 | 43 | 14 | 100 | 36 |
| FM (multidisciplinary clinics, etc.) | | 7 | 33 | 13 | 60 | 14 | 29 | 50 | 22 |
| Vasculitis (ERN reference centres, etc.) | | 50 | 17 | 50 | 60 | 29 | 86 | 83 | 48 |
| Low-back pain (Spine clinics, etc.) | | 14 | 33 | 38 | 20 | 10 | 43 | 50 | 24 |
| Pain Clinic | | 36 | 50 | 13 | 20 | 14 | 43 | 67 | 30 |
| Spine Clinic | | 21 | 17 | 13 | 20 | 0 | 29 | 50 | 16 |
| National referral guidelines | | 29 | 17 | 13 | 20 | 10 | 71 | 100 | 30 |
| Local referral guidelines | | 29 | 17 | 0 | 20 | 19 | 86 | 100 | 33 |
| **GPs** | |  |  |  |  |  |  |  |  |
| Specialised care units | | 33 | 0 | 0 | 63 | 38 | 33 | 100 | 33 |
| RA (early arthritis clinics, etc.) | | 17 | 0 | 0 | 50 | 38 | 33 | 100 | 28 |
| SpA (early SpA clinics, etc.) | | 17 | 0 | 0 | 25 | 25 | 17 | 0 | 15 |
| Osteoporosis (FLS, etc.) | | 17 | 0 | 0 | 63 | 13 | 33 | 100 | 26 |
| FM (multidisciplinary clinics, etc.) | | 33 | 0 | 0 | 50 | 13 | 17 | 0 | 21 |
| Vasculitis (ERN reference centres, etc.) | | 33 | 0 | 0 | 25 | 13 | 0 | 100 | 15 |
| Low-back pain (Spine clinics, etc.) | | 33 | 0 | 0 | 63 | 13 | 0 | 100 | 23 |
| Pain Clinic | | 33 | 0 | 0 | 50 | 25 | 33 | 100 | 28 |
| Spine Clinic | | 33 | 0 | 0 | 50 | 13 | 17 | 100 | 23 |
| National referral guidelines | | 17 | 0 | 0 | 63 | 38 | 0 | 100 | 26 |
| Local referral guidelines | | 0 | 0 | 0 | 38 | 25 | 33 | 0 | 18 |
| **Public and patient perspective** |  |  |  |  |  |  |  |  |  |
| Pain units | Public | 63 | 18 | 38 | 50 | 75 | 50 | 59 | 51 |
|  | Private | 55 | 44 | 53 | 30 | 46 | 40 | 33 | 44 |
| Spine clinics | Public | 56 | 18 | 57 | 42 | 20 | 43 | 63 | 45 |
|  | Private | 45 | 22 | 53 | 50 | 23 | 60 | 33 | 42 |
| Early arthritis clinics | Public | 25 | 45 | 29 | 25 | 33 | 30 | 59 | 34 |
| Multidisciplinary clinics | Public | 60 | 36 | 33 | 33 | 40 | 29 | 47 | 39 |
|  | Private | 55 | 56 | 24 | 30 | 54 | 20 | 33 | 38 |
| Self-appointment to rheumatology | Public | 25 | 64 | 33 | 8 | 13 | 25 | 13 | 25 |
|  | Private | 27 | 78 | 71 | 20 | 54 | 45 | 33 | 49 |
| Imaging services (X-rays, ultrasound, MR...) | Public | 94 | 91 | 100 | 83 | 94 | 75 | 100 | 90 |
|  | Private | 91 | 100 | 94 | 70 | 86 | 40 | 56 | 79 |

Cells represent the percentage responding affirmatively per country. Abbreviations: RA, rheumatoid arthritis; SpA, Spondyloarthritis; FLS, Fracture Liaison Services; FM, Fibromyalgia; ERN, European Reference Networks.

# Table S5. Type of patients commonly seen in rheumatology.

|  | **DE** | **HE** | **HU** | **NE** | **ES** | **SE** | **UK** | **Total** |
| --- | --- | --- | --- | --- | --- | --- | --- | --- |
| Inflammatory rheumatic diseases | 93 | 100 | 100 | 100 | 90 | 100 | 100 | 96 |
| Osteoarthritis | 36 | 83 | 63 | 80 | 81 | 14 | 33 | 58 |
| Gout | 64 | 83 | 75 | 100 | 81 | 57 | 50 | 73 |
| Soft tissue pain | 36 | 83 | 63 | 80 | 76 | 29 | 33 | 58 |

Cells represent the percentage responding affirmatively per country.

# Table S6. Level of perceived organisation of the health system.

| **How well is your health system organised?** | **DE** | **HE** | **HU** | **NE** | **ES** | **SE** | **UK** | **Total** |
| --- | --- | --- | --- | --- | --- | --- | --- | --- |
| **Rheumatologists' perspective** |  |  |  |  |  |  |  |  |
| Very well | 0 | 0 | 0 | 0 | 0 | 43 | 0 | 5 |
| Well | 38 | 0 | 0 | 80 | 21 | 57 | 50 | 31 |
| Moderately | 62 | 67 | 50 | 20 | 58 | 0 | 33 | 47 |
| Poorly | 0 | 17 | 50 | 0 | 16 | 0 | 17 | 14 |
| Very poorly | 0 | 17 | 0 | 0 | 5 | 0 | 0 | 3 |
| Organisational score* | 338 | 250 | 250 | 380 | 295 | 443 | 333 |  |
| **GPs' perspective** |  |  |  |  |  |  |  |  |
| Very well | 20 | 0 | 0 | 57 | 0 | 25 | 0 | 19 |
| Well | 20 | 0 | 0 | 29 | 29 | 75 | 100 | 28 |
| Moderately | 40 | 0 | 0 | 14 | 57 | 0 | 0 | 22 |
| Poorly | 20 | 86 | 100 | 0 | 14 | 0 | 0 | 28 |
| Very poorly | 0 | 14 | 0 | 0 | 0 | 0 | 0 | 3 |
| Organisational score* | 340 | 186 | 200 | 443 | 314 | 425 | 400 |  |

Cells represent the percentage of clinicians who marked the level per country.

*Calculated as a weighted sum of scores, with “very well [organised]” responses multiplied by 5 and “very poorly” by 1, and all other scores in between (See Methods).

# Table S7. Direct communication between healthcare levels.

| **Direct communication Primary Care-Rheumatology** | **DE** | **HE** | **HU** | **NE** | **ES** | **SE** | **UK** | **Total** |
| --- | --- | --- | --- | --- | --- | --- | --- | --- |
| Rheumatologists who answer affirmatively | 29 | 0 | 20 | 0 | 64 | 86 | 67 | 47 |
| GPs who answer affirmatively | 0 |  |  | 100 | 67 | 100 | 100 | 77 |

Cells represent the percentage responding affirmatively per country.
