# Supplementary material for: The journey of patients with musculoskeletal complaints in Europe: a cross-sectional European survey
Source: Rheumatol Int. 2025 Apr 18;45(5):107. doi: 10.1007/s00296-025-05863-x (PMC12008060; doi:10.1007/s00296-025-05863-x)
Supplement: Supplementary file 2 — Supplementary Material 2 [file 296_2025_5863_MOESM2_ESM.docx]

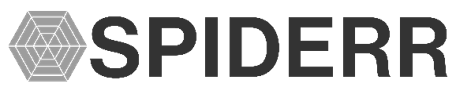


The Journey of Patients with Musculoskeletal Problems in Europe (GPs)

# Thank you for your interest in participating in our study.

We are investigating the journeys of people with musculoskeletal complaints in Europe, how they contact the health system, if they contact at all, how the experience is with the diagnosis and the treatment, and what can be improved.

This is part of a Horizon-funded project, SPIDeRR, to improve the identification, diagnosis, and treatment of people with musculoskeletal complaints and rheumatic and musculoskeletal diseases in Europe using AI.

The survey will take around **7 minutes** to complete.

Please answer the best you can. There are no right or wrong answers.

We will not ask you for personal details that could identify you.

If you agree to participate, please click on the **Next** button.


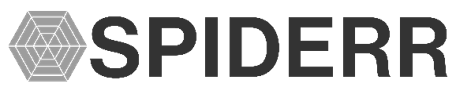


Sociodemographic questions

The Journey of Patients with Musculoskeletal Problems in Europe (GPs)

# First of all, please answer these questions so that we can describe the people who responded to the survey.

## * The country you live in is…?


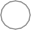
 Germany
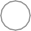
 Greece
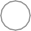
 Hungary


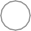
 Netherlands
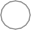
 Spain


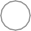
 Sweden
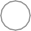
 UK


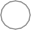
 Other (please specify)

## Do you (mainly) work for your country's...?


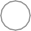
 Public system
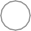
 Private system


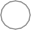
 Other (please specify)

## You work setting is...?


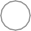
 Urban
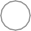
 Rural


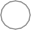
 Other (please specify)

## You practice...?


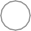
 Alone (solo practice)


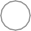
 In a group/centre, sharing the pool of patients


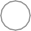
 In a group/centre, not sharing the pool of patients
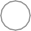
 Other (please specify)


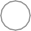
I do not attend patients currently

## Your gender is…?


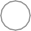
 Male
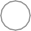
 Female


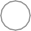
 Non-binary
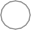
 Others

## You have been working as a general practitioner/family doctor…?


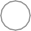

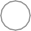
 less than 5 years
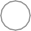
 5 to 20 years

more than 20 years


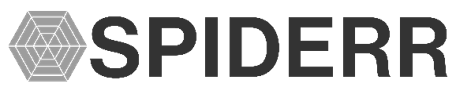


Access to healthcare

The Journey of Patients with Musculoskeletal Problems in Europe (GPs)

**For the following questions, think about your country in general. If you don't know, choose the answer "I don't know".**

**If you answer from a computer, you can move from option to option with the Tab key and then type y, n or I.**

**What services and professionals are available in your country for people with musculoskeletal complaints in the private and public sectors?**

## [Click all that apply]

Public Private


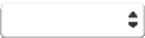

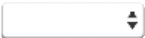

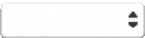

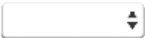

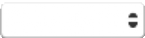

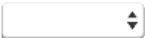

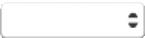

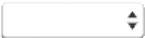

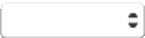

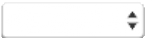

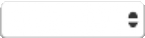

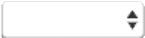

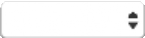

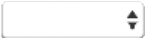

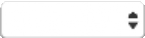

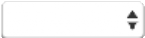

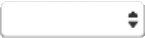

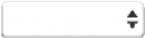

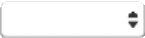

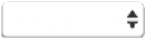


Primary care or Family doctors (GP) Orthopaedic Surgery/Traumatology Rheumatology

Physiotherapy Occupational therapy Rehabilitation Balneotherapy Specialist nurses

Psychologists (specialists in pain)

Sports doctors

Other services

# In your setting, is access to rheumatology mandatorily provided by GPs?


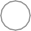

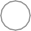
 Yes
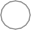
 No

It depends (please specify)

# In your setting, have GPs access to these diagnostics?

## [Click all that apply]


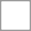
 Rheumatoid factor


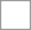
 ACPA (anti-CCP antibodies)
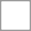
 HLA B27


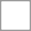
 ANA (antinuclear antibodies)


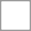
 Acute phase reactants (ESR, C-RP)
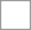
 Hands and feet X-Rays

Sacroiliac MRI

# In your setting, can GPs prescribe these treatments?

## [Click all that apply]


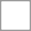
 Methotrexate
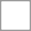
 Salazopyrine


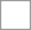
 Hydroxychloroquine
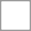
 Glucocorticoids


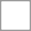
 Physiotherapy (referral) Psychology (referral)

Comments


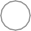

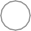

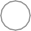

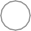

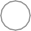

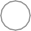

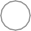

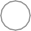

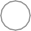


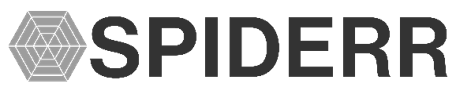


Care pathways

The Journey of Patients with Musculoskeletal Problems in Europe (GPs)

# A care pathway is an official process that guides patients and healthcare providers through diagnosis, treatment, and management. It includes tailored interventions like assessments, medication, therapy, or referrals.

**How likely is it that a person with musculoskeletal complaints will try to get a diagnosis in your country via the following?**

## [Click all that apply]


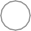

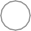

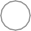

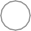

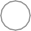

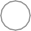
Very unlikely Could be Very unlikely


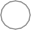

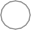

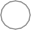


Searching the internet

Primary care or Family doctors (GP)


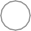

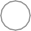

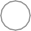


Orthopaedic Surgery/Traumatology

Rheumatology


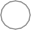

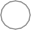

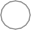


Physiotherapy

Rehabilitation


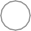

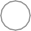

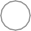


Balneotherapy

Specialist nurses


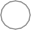

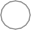

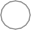


Psychologists (specialists in pain)

Pain units


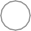

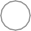

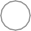


Spine clinics

Sports doctors


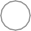

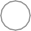

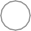


Occupational medicine

Comments

A **specialised care unit** is a team of specialists, like rheumatologists, nurses, and physical therapists, who diagnose and treat specific conditions, such as back pain or rheumatoid arthritis. They provide personalised care, including diagnostic tests, medication management, physical therapy, and education, to improve patients' quality of life.

# Are specialised care units or health pathways established for rheumatic or musculoskeletal diseases in your setting?

Yes No

I don’t know

The Journey of Patients with Musculoskeletal Problems in Europe (GPs)

# What specific pathways are there in your country?

## [Mark all that apply]

Rheumatoid arthritis (early arthritis clinics, etc.) Spondyloarthritis (early SpA clinics, etc.)

Osteoporosis (Fracture Liaison Services, etc.) Fibromyalgia (multidisciplinary FM clinics, etc.) Vasculitis (ERN reference centres, etc.)

Low-back pain (Spine clinics, etc.) Pain clinic

Spine clinic

National referral guidelines Local referral guidelines

Comments

IT systems

The Journey of Patients with Musculoskeletal Problems in Europe (GPs)

# Please refer to the informatics and codification rules in your setting

**What type of coding is used in Primary Care?**

ICD CPT

HCPCS

Other (please specify)

None or I don't know

# Does your IT system allow you to communicate (speaking directly to the specialist and getting feedback) with rheumatology?

Yes No

Comments

Your opinion

The Journey of Patients with Musculoskeletal Problems in Europe (GPs)

# Answer what best fits what you think.

## In your country, how relevant is the support from the community and social environment when seeking help?

Very relevant Quite relevant

Moderately relevant Slightly relevant

Not relevant at all

## How knowledgeable do you feel are family doctors about rheumatic diseases?

Very knowledgeable Knowledgeable

Moderately knowledgeable Not very knowledgeable Not knowledgeable at all

## How well organised do you find your healthcare system?

Very well Well

Moderately Poorly

Very poorly

## How difficult is it to get a referral to rheumatology?

Very easy Easy

Neither easy nor difficult Difficult

Very difficult

I have no idea

## On average, how long does it take to get a diagnosis of a rheumatic disease?

Less than 1 month 1-3 months

4-6 months

7-12 months

More than 12 months I have no idea

## When patients are referred to rheumatologists, are they always seen by the same doctor? (continuity of care)

Yes, always

Most of the time Sometimes

Rarely Never

I don't know

## Do you want to tell us something about your health system that is important for the patient journey, but we did not ask?

This is all, thank you very much!

If you would like to know the results of this survey or would like to be invited to other parts of this research, please write to [loreto.carmona@inmusc.eu](mailto:loreto.carmona@inmusc.eu)

If you want to know more about SPIDeRR go to <https://spiderr-project.eu/>

SPIDeRR has received funding from the Horizon Europe programme under grant agreement 101080711
