# Supplementary material for: The journey of patients with musculoskeletal complaints in Europe: a cross-sectional European survey
Source: Rheumatol Int. 2025 Apr 18;45(5):107. doi: 10.1007/s00296-025-05863-x (PMC12008060; doi:10.1007/s00296-025-05863-x)
Supplement: Supplementary file 3 — Supplementary Material 3 [file 296_2025_5863_MOESM3_ESM.docx]

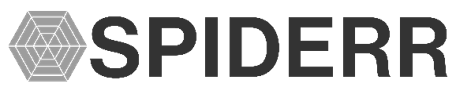


The Journey of People with Musculoskeletal Problems in Europe

# Thank you for your interest in participating in our study.

We are investigating the journeys of people with musculoskeletal complaints in Europe, how they contact the health system, if they contact at all, how the experience is with the diagnosis and the treatment, and what can be improved.

Even if you don’t have a rheumatic disease or any musculoskeletal complaints, you may have contacted the health system in your country at some point, or you may guess how it works. We also need that information: how people see the health system before they even need to use it for a rheumatic problem.

In other words, we want to know the critical aspects and touchpoints of the European health systems concerning the identification, diagnosis, and treatment of people with musculoskeletal complaints and rheumatic and musculoskeletal diseases, even before they appear.

The survey will take around **6 minutes** to complete.

Please answer the best you can. There are no right or wrong answers.

We will not ask you for personal details that could identify you.

If you agree to participate, please click on the **Next** button.


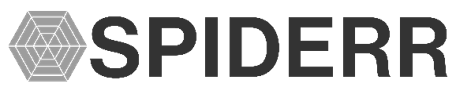


Sociodemographic questions

The Journey of People with Musculoskeletal Problems in Europe

# First of all, please answer these questions so that we can describe the people who responded to the survey.

## * The country you live in is…?


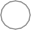
 Germany
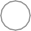
 Greece
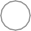
 Hungary


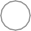
 Netherlands
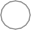
 Spain


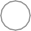
 Sweden
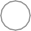
 UK


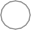
 Other (please specify)

## * Do you have a rheumatic disease?


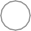
 Yes, I have been diagnosed with a rheumatic or musculoskeletal disease
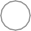
 Maybe, because I have some symptoms


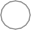
 No, I don't think so

If you have a rheumatic disease, can you tell us which one/s?

## Your age is…?


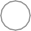
 less than 30


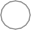
 30 to 50


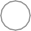
 more than 50

## Your gender is…?


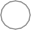
 Male
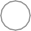
 Female


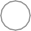

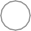
 Non-binary Others

## What do you perceive as your socioeconomic level compared to other people in your country? Consider your income, professional achievement, and level of education.


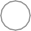
 Very low
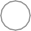
 Low


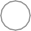

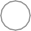
 Medium
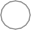
 High

Very high


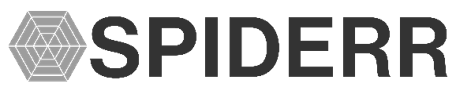


Access to healthcare

The Journey of People with Musculoskeletal Problems in Europe

**For the following questions, think about your country in general. If you don't know, choose the answer "I don't know".**

**If you answer from a computer, you can move from option to option with the Tab key and then type y, n or I.**

**If you had a problem with your joints, muscles, or bones, would you have access in your country to these professionals?**

## [Click all that apply]

Public Private


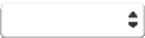

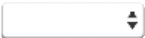

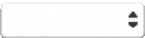

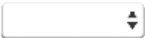

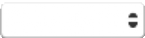

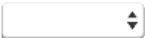

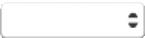

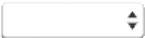

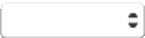

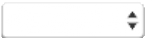

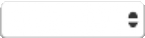

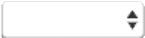

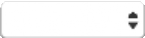

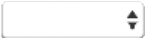

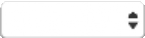

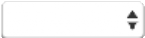

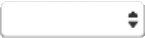

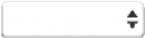

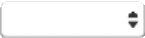

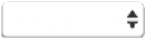

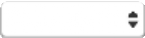

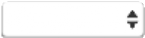

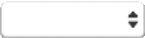

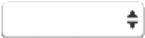

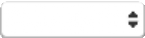

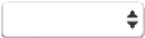

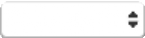

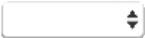

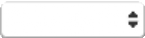

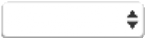

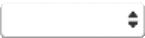

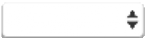


Primary care or Family doctors (GP) Orthopaedic Surgery/Traumatology Rheumatology

Physiotherapy Occupational therapy Rehabilitation Balneotherapy Specialist nurses

Psychologists (specialists in pain) Pain units

Spine clinics Sports doctors

Early arthritis clinics Multidisciplinary clinics

Self-appointment to rheumatology

Imaging services (radiology, ultrasound, MR...)

Comments


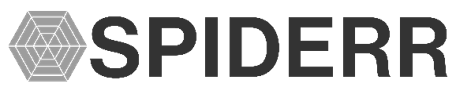


Seeking help

The Journey of People with Musculoskeletal Problems in Europe

# If you had symptoms (like pain or fatigue), how likely would you be to try these?

I

don't know what

Very

Somewhat

Very

this


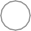

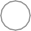

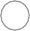

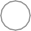

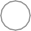

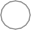

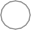

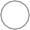

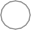

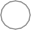
likely likely Unlikely unlikely is


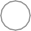

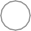

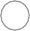

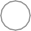

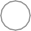


Search the internet directly (Google or similar search engine)

Use a symptom checker


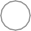

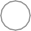

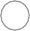

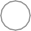

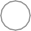


Use AI, like ChatGPT

Check my social media (TikTok, Facebook, Instagram, etc.)


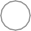

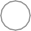

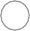

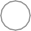

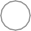


Go to my Primary care or Family doctor (GP)

Go directly or try to get an appointment with Orthopaedic Surgery/Traumatology


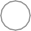

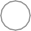

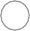

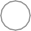

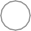


Go directly or try to get an appointment with Rheumatology


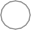

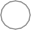

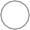

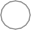

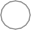

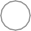

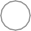

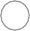

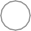

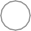

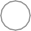
Go to Physiotherapy

Go to Rehabilitation

Go to Balneotherapy

Go to a Pain unit

Go to a Spine clinic

Go to an Occupational therapist Other (please specify)

Go to a Sports doctors

Your opinion

The Journey of People with Musculoskeletal Problems in Europe

# Answer what best fits what you think.

## In your country, how relevant is the support from the community and social environment when seeking help?

Very relevant Quite relevant

Moderately relevant Slightly relevant

Not relevant at all

## How well organised do you find your healthcare system?

Very well Well

Moderately Poorly

Very poorly

## * In your country, how important is the general practitioner/family doctor in getting a referral to a specialist?

Very important Important

Moderately important Slightly important

Not important at all

## How difficult is it to get a referral to rheumatology?

Very easy Easy

Neither easy nor difficult Difficult

Very difficult I don't know

# If you have a rheumatic disease

## How long did it take you to get the diagnosis since you started with symptoms?

Less than 1 month 1-3 months

4-6 months

7-12 months

More than 12 months I do not have a diagnosis

## Do you want to tell us something about your health system that you think is important but we did not ask?

This is all, thank you very much!

If you would like to know the results of this survey or would like to be invited to other parts of this research, please write to [loreto.carmona@inmusc.eu](mailto:loreto.carmona@inmusc.eu)

If you want to know more about SPIDeRR go to <https://spiderr-project.eu/>

SPIDeRR has received funding from the Horizon Europe programme under grant agreement 101080711
